# Social Network Size and Cognitive Functioning in Middle-Aged Adults: Cross-Sectional and Longitudinal Associations

**DOI:** 10.1007/s10804-016-9248-3

**Published:** 2016-11-15

**Authors:** Daniel Eriksson Sörman, Michael Rönnlund, Anna Sundström, Margareta Norberg, Lars-Göran Nilsson

**Affiliations:** 1 Department of Psychology, Umeå University, 901 87 Umeå, Sweden; 2 Centre for Demographic and Ageing Research (CEDAR), Umeå University, Umeå, Sweden; 3 Department of Public Health and Clinical Medicine, Umeå University, 901 87 Umeå, Sweden; 4 Aging Research Center (ARC), Karolinska Institutet, 113 30 Stockholm, Sweden; 5 Umeå Center of Functional Brain Imaging, Umeå University, 901 87 Umeå, Sweden

**Keywords:** Cognitive functioning, Social network, Longitudinal, Cross-sectional, Cognitive reserve

## Abstract

The objective of the present study was to examine relations between social network size and three cognitive abilities (episodic memory, semantic memory, visuospatial ability) in middle-aged adults. We analyzed cross-sectional data on social network size and cognitive functioning that were available for 804 participants aged 40–60 years. In addition, we examined 5- and 10-year follow-up measurements of cognitive functioning that were available for 604 and 255 participants, respectively. Cross-sectional analyses revealed a positive association between social network size and each of the three cognitive abilities. Baseline network size was positively related to 5-year changes in semantic memory, and to 10-year changes in semantic as well as episodic memory, but was unrelated to changes in visuospatial performance. A minor portion of the sample (*n* = 131) had 10-year follow-up data on network size. Cross-lagged panel correlations revealed that baseline network size was associated with follow-up measurement in cognitive functioning (episodic memory, semantic memory), whereas baseline cognitive performance was unrelated to future network size. Together, the results demonstrate a small but positive relation between network size and declarative memory abilities, in line with models proposing a cognitive reserve built up by factors such as the increased cognitive stimulation associated with a more extensive social network.

## Introduction

A number of studies have shown that engagement in social relationships is negatively related to mortality and positively related to a variety of health outcomes (Barger 2013; Tay et al. 2013). Available evidence also indicates a positive association between social relationships and cognitive functioning in old age (e.g., Beland et al. 2005; Ertel et al. 2008; Fratiglioni et al. 2004; Lövdén et al. 2005; Zunzunegui et al. 2003).

Such positive associations between social relationships and cognitive function have been taken to suggest that social relationships reduce cognitive decline. One possibility is that social activity puts cognitive demands on many cognitive components or processes such as memory, attention, inhibition, and adaption to the perspectives and desires of other (Ybarra et al. 2008). These demands on cognitive functioning may in turn build up a reserve capacity that allows for more efficient use of neural networks, thereby minimizing age-related decline (Scarmeas and Stern 2003; Stern 2002). Findings from a longitudinal study using postmortem examinations—and demonstrating that cognitive functions remained higher for individuals with larger social network size even in the presence of severe levels of Alzheimer’s disease pathology (Bennett et al. 2006)—would seem to support these assumptions, although a reversed causal influence (i.e., lowered social engagement as a consequence of cognitive decline) must be considered (Hertzog et al. 1999).

*Social relationships* is a broad term used to refer to the functional as well as structural aspects of interpersonal relationships (Holt-Lunstad et al. 2010). The term *functional aspects* refers to qualitative characteristics of social relationships, for example, the availability of informational or emotional support. Measures that reflect the *structural aspects* of social relationships include marital status, the size of a person’s social network, the frequency of contact with others, or participation in social activities. The present focus was on social network size, which may thus be regarded as a structural measure. However, questions commonly used to estimate social network size (e.g., “How many friends do you have?” and “How many relatives do you feel close to?”; Cohen et al. 1997) presuppose a certain level of (positive) emotional involvement. Moreover, in a conceptual model proposed by Berkman et al. (2000), social networks were hypothesized to promote health through a variety of mechanisms (e.g., provision of social support, social influences, social engagement, and access to material goods and resources; cf. “social capital” as used by Bourdieu 1986). Thus, whereas simple network indices may be thought to mainly reflect a quantitative aspect of social relationships, they probably pick up some variability with regard to emotional/functional (even sociocultural) aspects of social relationships as well. Regardless of this matter, network size may be regarded as providing a (rough) proxy measure of the degree of “social stimulation” an individual is likely to experience, which gave us a foundation for examining potential links to cognitive functioning in the present study.

The relation between social network size and cognitive functioning has been addressed in a few studies on older adults. Some of these studies have demonstrated that having a larger network is associated with having a higher level of cognitive performance and/or reduced time-related decline using measures of global cognitive cognition, such as MMSE (Holtzman et al. 2004), the Short Portable Mental Status Questionnaire (Bassuk et al. 1999), or a composite score based on two memory measures (MMSE and a measure of processing speed; Barnes et al. 2004). However, other studies have failed to detect any such association between network size and cognitive performance (see Glei et al. 2005; Seeman et al. 2001a, b).

Whereas a growing number of studies have focused on the relation between social resources and cognitive functioning in older adults, little attention has been paid to middle-aged adults (e.g., Richards et al. 2003; Seeman et al. 2011; Singh-Manoux et al. 2003; Ybarra et al. 2008). In fact, we are aware of only one prior study that focused on network size and cognitive performance based on a sample with a mean age below 60 years (*M* = 47.3 at baseline; Green et al. 2008). The results indicated a significant relation between network size and cognitive performance in the cross-sectional analyses, but no association with changes in performance from baseline to a 10-year follow-up. Like several other studies in the literature focusing on the elderly, Green et al. (2008) used single indicators (MMSE and word recall) of the cognitive constructs, which limits the conclusions that may be drawn from the results.

The relative absence of studies targeting cognitive performance in midlife in relation to social network size is noteworthy, given the fact that this period is characterized by changes in social networks, including a steady decrease in personal network and friendship network (Wrzus et al. 2013) and, in particular, given evidence of the onset of a decline in “fluid” cognitive abilities (e.g., speed of processing, visuospatial ability) in late middle-age (e.g., Rönnlund and Nilsson 2006a; Rönnlund et al. 2005; Schaie 1994). Hence, it is important to identify factors (e.g., social) that may account for interindividual differences in onset of this decline.

Given indications that social network size is related to cognitive functions in old age and the relative paucity of studies targeting middle-aged adults, the objective of the present study was to examine the relation between social network size and cognitive performance in a population-based sample of adults aged 40–60 years. We examined relations between network size and cognitive performance in cross-sectional analyses and based on data from 5- to 10-year follow-up measurements of cognitive performance. In contrast to prior studies, which have often used rather crude measures of cognitive functioning, the present study involved comprehensive measures of episodic memory [including retrieval (recall/recognition) of personally experienced events], semantic memory (reflecting knowledge and fluency tasks; Nyberg et al. 2003), and visuospatial ability, with the specific aim being to investigate the generalizability of potential associations with social network size across these abilities. A variety of potentially confounding variables (e.g., demographic, health, and lifestyle factors) were taken in account. Previous studies based on the sample used in the present study have demonstrated that the episodic memory measures (particularly the recall measures) are highly age sensitive and that aspects of semantic memory, including speedy retrieval of knowledge, deteriorate in old age (Rönnlund et al. 2005), as does the visuospatial ability measure used here (WAIS-R Block Design; Rönnlund and Nilsson 2006a). Additionally, studies have revealed interindividual differences in longitudinal change for both the measures of memory and of visuospatial ability (e.g., Lövdén et al. 2004; Rönnlund and Nilsson 2006a). It is of theoretical interest to note that the latter measure is nonverbal, whereas the episodic memory and particularly the semantic memory tests draw on verbal/communicative skills. Considering the theoretical notion that the influence of social relationships on cognitive performance is based on a common factor (e.g., improved mood), one might expect similar associations across the measures. To the extent that social contacts serve to train/maintain verbal (memory) skills, the effects might, by contrast, be expected to be largest for semantic memory and smallest for the measure of visuospatial ability.

## Method

### Study Population

We included data drawn from two separate sources: the Betula prospective cohort study (Nilsson et al. 1997; 2004) and the Västerbotten Intervention Programme (VIP; Norberg et al. 2010). These datasets are individually linked within the Linnaeus database (Malmberg et al. 2010) at the Centre for Demographic and Ageing Research (CEDAR) at Umeå University, Sweden. The Linnaeus database, designed for both cross-sectional and longitudinal studies, was developed to provide interdisciplinary research and includes information from several sources of Swedish register data.

The Betula study started in 1988 in Umeå in the county of Västerbotten, Sweden, and is an ongoing study of aging, memory, and health. The aims of the study are to examine how health and memory develop over the lifespan, to detect preclinical signs of dementia, and to evaluate premorbid memory function. The participants in the Betula study were selected from the population registry of Umeå Municipality using stratified (age, sex) random sampling. Data have been collected at five test waves—1988–1990 (T1), 1993–1995 (T2), 1998–2000 (T3), 2003–2005 (T4), and 2008–2010 (T5)—and involved 6 samples ranging in age from 25 to 90 years at the time of inclusion. The participants were assessed on two occasions at each test wave, about 1 week apart, with the first session focusing on health examination and the second on cognitive functioning (for a detailed description of the Betula study, see Nilsson et al. 1997; 2004).

The VIP is an ongoing population-based intervention that began in 1985. Its purpose is to reduce cardiovascular diseases. Since 1995, all inhabitants within the County of Västerbotten who are 40, 50 and 60 years have been invited to participate. The examination, carried out on a single occasion, included a medical inspection and a questionnaire concerning health, lifestyle factors, and life situation. For each participant, a health profile was constructed and the outcomes were discussed together with a nurse; the aim was health promotion. A more detailed description of VIP can be found elsewhere (Norberg et al. 2010).

Data from test occasions in 1993–1995, 1998–2000, and 2003–2005 were considered in the present study, because linked data regarding both questions of social networks (VIP) and cognitive information (Betula) were available for these test waves.

### Participants

We included participants with data available from both Betula (sample 1–3) and VIP that were collected no more than 12 months apart (*n* = 842). For a few participants, data on the targeted variables were missing, and hence, they were excluded. The variables and number of excluded participants were: social network (*n* = 14), cognition (*n* = 4), education (*n* = 12), physical exercise (*n* = 3), subjective health (*n* = 4), and use of alcohol (*n* = 1). This left a sample of 804 for the present analyses.

**Fig. 1:**
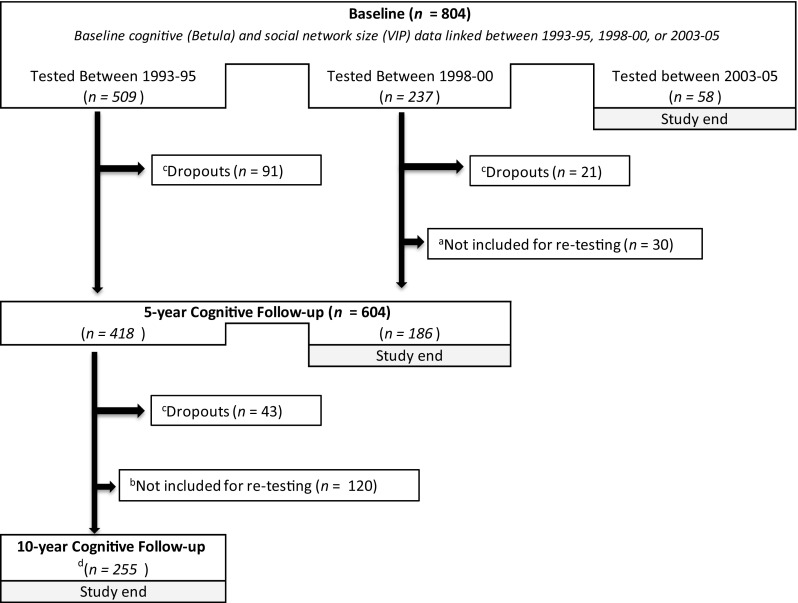
Design and flowchart of the study. Participants were tested every fifth year in the Betula study and every tenth year in VIP. ^a^Due to the study design, some participants from Sample 3 in the Betula study were not retested. ^b^Due to the study design, participants from Sample 3 in Betula study were not retested. ^c^Dropouts are participants who were deceased or did not want to participate at the follow-up measurement. ^d^For some participants (*n* = 131), follow-up information regarding social network size was also available

At the time of the 5-year cognitive follow-up measurement, data were available for 604 participants, and for the 10-year follow-up, cognitive data were available for 255 participants. For a minor proportion (*n* = 131), data on both cognitive function and social network size at 10-year follow-up were available. The design and flow chart of the study are presented in Fig. 1.

### Measures (Database in Brackets)

#### Episodic Memory (Betula)

Six tests were used to measure episodic memory ability (for a detailed description, see Rönnlund and Nilsson 2006b; for evidence of loadings of the measures on a common episodic factor, see Nyberg et al. 2003). These were: (1) free oral recall of 16 verb–noun sentences enacted in the study phase, (2) free oral recall of 16 sentences encoded without enactment, (3 & 4) category-cued recall of nouns from the study list of enacted and non-enacted sentences using eight semantic categories, (5) recall of 12 nouns presented at a pace of 2 s per item, with paced (2 s/item) free recall, and (6) activity recall, a test in which the participants were asked to recall (describe) as many of the tests they had performed during the test session as possible. The total number of recalled tasks served as the score. A unit-weighted (z) episodic memory composite score was computed based on the six tests. The test–retest coefficient for the composite score was *r* = .752 (*p* < .01) and *r* = .734 (*p* < .01) for the 5-year and 10-year follow-up, respectively.

#### Semantic Memory (Betula)

Four indicators of semantic memory (for evidence of loadings of the measures on a common semantic factor, see Nyberg et al. 2003) were included. Three of them were fluency tasks, requiring generation of as many words as possible during 1 min. The restrictions were as follows: (1) words with the initial letter A (except names), (2) words beginning with the letter M and containing five letters (not names), and (3) professions beginning with the letter B. Additionally, the number of correctly identified synonyms on a 30-item multiple-choice test (Dureman 1960) was included as a measure of semantic memory. A semantic composite (z) score was generated based on the four indicators. The stability coefficient for the semantic composite score was *r* = .785 (*p* < .01) for the 5-year follow-up and *r* = .751 (*p* < .01) for the 10-year follow-up.

#### Visuospatial Ability (Betula)

The WAIS-R Block Design Test (Wechsler 1981) was used as a measure of visuospatial ability. In this test, the participants are required to duplicate prespecified patterns using four or nine bicolored blocks presented in ascending order of difficulty. The maximum number of patterns to solve is nine. Based on the number of trials and time to complete the designs, the participants obtain summary (raw) scores, which for the present purposes were transformed into z-scores. Cronbach’s *α* of .82 has been reported for this test in a Betula sample (Rönnlund and Nilsson 2006a). The stability coefficient for the visuospatial composite score was *r* = .783 (*p* < .01) for the 5-year follow-up and *r* = .764 (*p* < .01) for the 10-year follow-up.

#### Social Network Size (VIP)

Information about network size was collected as part of a questionnaire constructed to measure social relationships. Four questions were used as indicators of social network size: (1) “How many persons do you know and have contact with who have the same interests as you?” (2) “How many persons, whom you know, do you see or talk with during a regular week?” (3) “How many friends do you have who can come home to you at any time and feel at home?” (4) “How many are there, in your family and among your friends, to whom you can talk freely without reflection?” For each of the questions, the participants were requested to indicate the relevant number on a scale including the following response alternatives: nobody (coded as 0), 1–2 persons (1), 3–5 persons (2), 6–10 persons (3), 11–15 persons (4), and more than 15 persons (5). A composite score for network size was computed as the sum scores of the four questions (max = 20; Cronbach’s *α* = .77)

#### Covariates

Additional factors included (Betula) in the analyses were: age, gender (female = 1), years of education, and alcohol use (yes = 1). A subjective health rating (VIP) was included ranging from “very good,” “quite good,” “reasonably,” “rather poor” to “poor.” For the analyses, rather poor and poor were coded as 0; reasonably, quite good, and very good were coded as 1. Furthermore, an index of depressive symptoms (Betula) was included. This index was computed as the sum of six self-reported symptoms: (1) feeling anxious, (2) loss of appetite, (3) often feel dispirited, (4) often feel lonely, (5) sleeping problems, and (6) fatigue. This index has a Cronbach’s *α* of .63 and correlates reasonably well (*r* = .57; Rönnlund et al. 2015) with the Swedish version of the CES-D Scale (Radloff 1997). Finally, for estimation of physical exercise (VIP), the participants rated frequency of participation in exercise “within the past three months, wearing an exercise outfit, with the purpose of improving my physical status or feeling good” on a ordinal scale ranging from “never,” “now and then—not regularly,” “once a week,” “2–3 times a week,” to “more than 3 times a week.” For the analyses, a dummy was generated to indicate low activity (“never,” “now and then—not regularly”) coded as 0, and high activity (“once a week” or more) coded as 1.

### Statistical Analysis

Baseline differences between returnees and non-returnees on the demographic variables were analyzed using Student’s *t* test for the continuous variables and Chi-square for the categorical variables. Next, zero-order correlations between all the variables included in the analyses were computed. To investigate the associations between social network size and cognitive functioning, we used hierarchical multiple regression analyses, with the memory composites (episodic, semantic, and visuospatial) as dependent variables. In Step 1, we controlled for age, gender, and years of education. In Step 2, subjective health, depressive symptoms, physical exercise, and alcohol use were added. In Step 3, the sum score for social network size was entered. In the longitudinal analyses, the follow-up memory composites were used as dependent variables. We controlled for cognitive performance at the first test wave in Step 1, followed by the steps described above.

## Results

The sample included 804 participants at baseline. At the 5-year follow-up, cognitive data were available for 604 participants, and at the 10-year follow-up for 255 participants. Participant characteristics, as a function of follow-up period, are provided in Table 1.

**Table 1:** Characteristics of participants in both the cross-sectional and the longitudinal analyses

Baseline characteristic	Cross-sectional	Five-year follow-up		Ten-year follow-up	
	Sample	With data	Without data	With data	Without data
	(*n* = 804)	(*n* = 604)	(*n* = 200)	(*n* = 255)	(*n* = 549)
Age, *M* (SD)	52.17 (7.59)	52.28 (7.38)	51.85 (8.21)	49.92 (8.27)	53.21 (7.01)***
Years of education, *M* (SD)	11.94 (3.89)	11.88 (3.91)	12.11 (3.87)	12.22 (4.00)	11.80 (3.84)
Female (%)	55.7	55.3	57.0	56.5	55.4
Subjective health—fairly < (%)	94.4	94.9	93.0	95.7	93.8
Physical exercise—once a week < (%)	34.1	33.8	35.0	38.4	32.1
Alcohol use (%)	88.8	88.2	90.5	90.2	88.2
Depressive symptoms, *M* (SD)	.79 (1.08)	.70 (1.02)	1.04 (1.24)***	.67 (.99)	.84 (1.12)*
Network size, *M* (SD)	12.50 (3.82)	12.59 (3.87)	12.20 (3.69)	12.78 (3.78)	12.36 (3.84)

Baseline information for accessible participants with 5-year and 10-year cognitive follow-up is compared to those without cognitive follow-up

* *p* < .05; ** *p* < .01; *** *p* < .001

The mean age of the baseline sample (including 448 women and 356 men) was 52.17 (SD = 7.59). Compared to those (*n* = 200) included only at baseline, the returnees (*n* = 604) with 5-year cognitive follow-up data did not differ significantly with regard to background characteristics, except that they did exhibit fewer depressive symptoms, *t*(802) = −3.86, *p* < .001. The sample (*n* = 255) with 10-year cognitive follow-up data was younger, *t*(802) = −5.84, *p* < .001 and exhibited fewer depressive symptoms, *t*(802) = −2.06, *p* < .05, than the non-returnees did (*n* = 549).

Zero-order correlations between the variables are presented in Table 2. Age was negatively associated with episodic memory (*r* = −.304, *p* < .01) and visuospatial ability (*r* = −.284, *p* < .01), but to a lesser extent with semantic memory (*r* = −.135, *p* < .01). Network size was significantly correlated with all variables in the analyses, and most closely associated with depressive symptoms (*r* = −.192, *p* < .01), years of education (*r* = .188, *p* < .01), and the cognitive constructs (episodic, *r* = .160, *p* < .01; semantic memory, *r* = .178, *p* < .01; visuospatial ability, *r* = .168, *p* < .01).

**Table 2:** Correlations between variables used in the hierarchical multiple regression analyses

*n* = 804	Age	Gender	Years of education	Subjective health	Depressive symptoms	Physical exercise	Alcohol use	Episodic memory	Semantic memory	Visuospatial ability	Network size	
Age	–											
Gender	.016	–										
Years of education	−.339**	−.004	–									
Subjective health	−.066	−.119**	.086*	–								
Depressive symptoms	.034	.159**	.021	−.233**	–							
Physical exercise	−.171**	.039	.188**	.015	−.025	–						
Alcohol use	−.158**	−.102**	.165**	.068	−.070*	.039	–					
Episodic memory	−.304**	.196**	.475**	.064	−.021	.140**	.114**	–				
Semantic memory	−.135**	.143**	.490**	.074*	−.004	.070*	.105**	.520**	–			
Visuospatial ability	−.284**	−.104**	.341**	.067	.045	.101**	.072*	.406**	.361**	–		
Network size	−.108**	−.089**	.188**	.108**	−.192**	.081*	.092**	.160**	.178**	.168**	–	

Coding for gender (female = 1), subjective health (reasonably < = 1), physical exercise (once a week < = 1), and alcohol use (yes = 1)

* *p* < .05; ** *p* < .01, (two tailed)

Next, we performed hierarchical multiple regression analyses of the cross-sectional data, with T1 performance on each of the three cognitive abilities as the regressor. The results are presented in Table 3.

**Table 3:** Cross-sectional analyses using hierarchical multiple regression with cognitive ability at time 1 as dependent variable

Predictor	Cross-sectional					
	Episodic memory		Semantic memory		Visuospatial ability	
	*ΔR* ^*2*^	*β*	*ΔR* ^*2*^	*β*	*ΔR* ^*2*^	*β*
Step 1	.287***		.261***		.159***	
Age		−.165***		.033		−.189***
Gender		.197***		.141***		−.102**
Education		.418***		.500***		.277***
Step 2	.006		.006		.002	
Subjective health		.030		.043		.014
Depressive symptoms		−.048		−.028		−.027
Physical exercise		.025		−.026		.021
Alcohol use		.037		.041		−.017
Step 3	.005*		.009**		.007*	
Network size		.074*		.099**		.088*
Total *R* ^*2*^	.298***		.275***		.168***	
*n*	804		804		804	

*ΔR* ^*2*^ R square change, *β* standardized beta

* *p* < .05; ** *p* < .01; *** *p* < .001

Regarding episodic memory, Step 1 of the regression, including the demographic variables, resulted in a significant increment in the explained variance, *R* ^*2*^ change = .287, *F* change (3, 800) = 107.44, *p* < .001, with higher age [*β* = −.165, *t*(800) = −5.21, *p* < .001] that was negatively associated with performance, whereas female gender [*β* = .197, *t*(800) = 6.59, *p* < .001] and more years of education [*β* = .418, *t*(800) = 13.18, *p* < .001] were positively related to performance. Entry of the variables in Step 2 (health and lifestyle factors) was not associated with an increment in the explained variance. Critically, network size, which was added in Step 3, was associated with a significant increment in the explained variance, *R* ^*2*^ change = .005, *F* change (1, 795) = 5.66, *p* < .05 [*β* = .074, *t*(795) = 2.38, *p* < .05]. Analyses with semantic memory as the dependent variable revealed a increment in explained variance for the demographic variables added in Step 1, *R* ^*2*^ change = .261, *F* change (3, 800) = 94.11, *p* < .001, where female gender [*β* = .141, *t*(800) = 4.63, *p* < .001] and education (*β* = .500, *t*(800) = 15.49, *p* < .001] were positively associated with performance. Social network size, added in Step 3, was also significantly associated, *R* ^*2*^ change = .009, *F* change (1, 795) = 9.78, *p* < .01 [*β* = .099, *t*(795) = 3.13, *p* < .01 for social network size]. Finally, for visuospatial performance, the demographic variables, *R* ^*2*^ change = .159, *F* change (3, 800) = 50.27, *p* < .001 [*β* = −.189, *t*(800) = −5.47, *p* < .001, for age; *β* = −.102, *t*(800) = −3.14, *p* < .01, for gender; *β* = .277, *t*(800) = 8.04, *p* < .001, for education], and network size, *R* ^*2*^ change = .007, *F* change (1, 795) = 6.74, *p* < .05 [*β* = .088, *t*(795) = 2.60, *p* < .05] explained a significant portion of the variance in performance.

Next, the longitudinal data were analyzed using cognitive performance on the follow-up measurement as the regressor, with control for baseline (T1) performance at Step 1. The longitudinal analyses regarding the 5-year follow-up are summarized in Table 4.

**Table 4:** Association between network size at baseline and cognitive change over five years using hierarchical multiple regression with cognitive performance at time 2 as dependent variable

Predictor	Five-year follow-up					
	Episodic memory		Semantic memory		Visuospatial ability	
	*ΔR* ^*2*^	*β*	*ΔR* ^*2*^	*β*	*ΔR* ^*2*^	*β*
Step 1	.566***		.616***		.613***	
Cognitive performance T1		.752***		.785***		.783***
Step 2	.033***		.012***		.024***	
Age		−.126***		.002		−.131***
Gender		.065*		.046		.018
Education		.122***		.118***		.063*
Step 3	.002		.004		.005	
Subjective health		.006		.000		−.002
Depressive symptoms		.004		.021		.048
Physical exercise		.005		.052*		−.045
Alcohol use		.044		.041		−.030
Step 4	.002^b^		.003*		.002	
Network size		.046^b^		.058*		.047
Total *R* ^*2*^	.603***		.635***		.645***	
*n*	604		604		604	

*ΔR* ^*2*^ R square change, *β* standardized beta

* *p* < .05; *** *p* < .001; ^b^ *p* = .088

In all cases, baseline cognitive performance, added in Step 1, was a significant predictor of future change in the explained variance in cognitive performance. For episodic memory, the demographic variables were significant (Step 2), *R* ^*2*^ change = .033, *F* change (3, 599) = 16.43, *p* < .001. Similar to the cross-sectional analysis regarding episodic memory, higher age (*β* = −.126, *t*(599) = −4.46, *p* < .001] was negatively associated with performance, whereas female gender [*β* = .065, *t*(599) = 2.43, *p* < .05] and more years of education [*β* = .122, *t*(599) = 3.97, *p* < .001] had a positive influence. However, neither the health and lifestyle factors (Step 3) nor network size (Step 4) was associated with *R* ^2^ increments, although baseline network size almost reached significance for change in explained variance, *R* ^*2*^ change = .002, *F* change (1, 594) = 2.93, *p* = .088 [*β* = .046, *t*(594) = 1.71, *p* = .088]. For semantic memory, both the demographic step, *R* ^*2*^ change = .012, *F* change (3, 599) = 6.41, *p* = < .001, and network size, *R* ^*2*^ change = .003, *F* change (1, 594) = 5.01, *p* = < .05, were associated with an increment in the explained variance. Predictors that were significantly associated were years of education [*β* = .118, *t*(599) = 3.87, *p* < .001], physical exercise [*β* = .052, *t*(595) = 2.03, *p* < .05], and network size, [*β* = .058, *t*(594) = 2.24, *p* < .05]. Regarding visuospatial ability, only the demographic step was significantly associated, *R* ^*2*^ change = .024, *F* change (3, 599) = 13.31, *p* < .001, with age [*β* = −.131, *t*(599) = −4.90, *p* < .001], and education [*β* = .063, *t*(599) = 2.32, *p* < .05], which were significant predictors above cognitive baseline performance.

Results from the analyses regarding the 10-year follow-up are presented in Table 5.

**Table 5:** Association between network size at baseline and cognitive change over 10 years using hierarchical multiple regression with cognitive performance at time 3 as dependent variable

Predictor	Ten-year follow-up					
	Episodic memory		Semantic memory		Visuospatial ability	
	*ΔR* ^*2*^	*β*	*ΔR* ^*2*^	*β*	*ΔR* ^*2*^	*β*
Step 1	.539***		.564***		.583***	
Cognitive performance T1		.734***		.751***		.764***
Step 2	.032***		.016*		.042***	
Age		−.156**		−.106*		−195***
Gender		.100*		.057		.000
Education		.053		.035		.042
Step 3	.010		.002		.003	
Subjective health		.040		−.032		.005
Depressive symptoms		006		−.033		.054
Physical exercise		.051		−.029		.017
Alcohol use		.087		.021		.006
Step 4	.007*		.010*		.001	
Network size		.088*		.104*		.032
Total *R* ^*2*^	.588***		.592***		.629***	
*n*	255		255		255	

*ΔR* ^*2*^ R square change, *β* standardized beta

* *p* < .05; ** *p* < .01; *** *p* < .001

As for the 5-year follow-up, cognitive performance at baseline was a significant predictor of performance at follow-up (Step 1). As concerns episodic memory, both the demographic step, *R* ^*2*^ change = .032, *F* change (3, 250) = 6.30, *p* = < .001, and network size, *R* ^*2*^ change = .007, *F* change (1, 245) = 4.13, *p* < .05, were significant [*β* = −.156, *t*(250) = −3.36, *p* < .01 for age; *β* = .100, *t*(250) = 2.36, *p* < .05 for gender; *β* = .088, *t*(245) = 2.03, *p* < .05 for network size]. For semantic memory, the demographic step, again, predicted a significant amount of the explained variance, *R* ^*2*^ change = .016, *F* change (3, 250) = 3.25, *p* < .05 [*β* = −.106, *t*(250) = −2.35, *p* < .05 for age]. Critically, network size was, once more, significantly associated with performance, *β* = .104, *t*(245) = 2.41, *p* < .05, and change, *R* ^*2*^ change = .010, *F* change (1, 245) = 5.78, *p* < .05. Finally, for visuospatial ability, only the demographic step, with age [*β* = −.195, *t*(250) = −4.56, *p* < .001] contributed increments in the explained variance, *R* ^*2*^ change = .042, *F* change (3, 250) = 9.32, *p* < .001, beyond baseline performance.

We were not able to perform analyses of covariation over time between network size and cognitive abilities due to the small number of participants (*n* = 131) with longitudinal data on both cognitive ability and network size. However, cross-lagged panel correlations were calculated. The results revealed that social network size at baseline was significantly associated with episodic memory at the 10-year follow-up (*r* = .219, *p* < .05), although episodic memory at baseline was unrelated to network size at the 10-year follow-up (*r* = −.005, *p* > .05). The pattern was similar for semantic memory. Baseline network was related to semantic memory at the 10-year follow-up (*r* = .209, *p* < .05), but a relation was not found for baseline semantic memory and future network size (*r* = .004, *p* > .05). For visuospatial ability, none of the cross-lagged panel correlations was significant.

## Discussion

The purpose of the present study was to examine the association between social network size and cognitive ability in a sample of middle-aged adults. The results from the cross-sectional analyses revealed significant associations between social network size and episodic memory, semantic memory, as well as visuospatial ability. These associations persisted following control for a number of potential confounders (e.g., demographic and health variables, physical exercise, alcohol use, and depressive symptoms). As regards changes in performance from baseline to longitudinal follow-up measurements, the associations remained significant for both semantic and episodic memory, except for 5-year changes in episodic memory that were only of borderline significance (*p* < .10) after control for covariates. Thus, we found that middle-aged participants with a more extensive social network not only performed better at baseline, but also showed better maintenance of both episodic and semantic memory over a 10-year period. Although the effect was small in terms of explained variance accounted for, we can see a trend toward an increased association from the 5- to the 10-year longitudinal follow-up, suggesting that the relation between social network and cognitive performance would have been larger had an extended time window been used.

The present results support previous studies on samples of older individuals (mean age >60 years; e.g., Bassuk et al. 1999; Holtzman et al. 2004), showing that social network size is related to cognitive functions such as various memory-related abilities. Furthermore, our results are consistent with studies reporting positive effects of other structural aspects of social relationships in middle age, such as engagement in social leisure activities (Richards et al. 2003; Singh-Manoux et al. 2003) or having a higher frequency of social contacts (Seeman et al. 2011; Ybarra et al. 2008).

Unlike the only prior study looking at social network size and cognitive performance in middle-aged adults (Green et al. 2008), we demonstrated that the association could also be generalized to longitudinal changes. Thus, our results suggest that social interaction may have long-term beneficial effects on various aspects of cognitive functioning, including episodic and semantic memory. Our results, showing a beneficial effect of having a larger social network size, are in line with the cognitive reserve hypothesis (Scarmeas and Stern 2003; Stern 2002). However, social network size may affect cognition through other pathways. Although the association between network size and memory changes emerged in the analyses controlling for depressive symptoms, we cannot rule out the possibility that larger networks offer support in coping with stressors in life (for a review, see Ozbay et al. 2007) or promote unmeasured health and lifestyle factors (see Lewis and Rook 1999) that may be beneficial to cognitive ability. Thus, studies should benefit from investigating the joint influence of social network size and other aspects of social relationships (e.g., emotional or instrumental support). Future studies should also consider the role of more specific ties (e.g., partner, children, or grandchildren). Obviously, the type of ties to consider could vary across the age range targeted in the present study (e.g., having grandchildren is much more likely at age 60 than at age 40).

However, it is not only the possible pathways through which social network size affects cognition that are difficult to identify. Social network size, per se, may be difficult, if not impossible, to disaggregate from other variables. It is highly plausible that social network patterns are influenced by, for example, mood (Uchino 2006) and/or health (Horgas et al. 1998; Lawton et al. 1987). As noted, we aimed to control for these factors in the present study, but we cannot exclude the possibility that social network size is related to other factors that we were not able to control for. Personality traits and/or attachment patterns, for example, are other factors that may influence what patterns of interaction with others look like (Pierce et al. 1997).

Whereas network size was positively associated with semantic and episodic memory, both in terms of baseline and future performance, a relation to visuospatial ability (Block Design Test) was only demonstrated in the cross-sectional analyses. As noted, the fact that the degree of association between network size and cognitive performance differed across the three cognitive abilities investigated—with the largest associations between network size and semantic memory and smallest between network size and visuospatial ability—is of potential theoretical interest, because these abilities may be considered to vary along a continuum from fluid (visuospatial ability) to mainly crystallized (semantic memory). One possible explanation for a stronger relation between network size and crystallized knowledge is that a larger social network is associated with more verbal skills training. Another potential explanation for our results is that positive associations are selectively observed for forms of declarative (i.e., episodic and semantic) memory, regardless of the verbal–nonverbal distinction. The cognitive stimulation provided by a larger network may have promoted growth and reduced negative changes in the neocortex and hippocampus, vital regions for episodic and semantic memory (Moscovitch et al. 2005), rather than the occipital and parietal regions of the brain that underlie visuospatial performance (Alichniewicz et al. 2012; Alivisatos and Petrides 1997). In line with this, it has been reported that environmental enrichment can promote some neurotrophic factors in regions important for episodic and semantic memory functioning. In their review, Valenzuela et al. (2007) reported that high levels of mental activity may be related to hippocampal neurogenesis. Although it has been reported that cognitive stimulation is also associated with brain metabolic activity, increased regional brain blood flow (Solé-Padullés et al. 2009), and more complex dendritic patterns (Valenzuela et al. 2007), such effects are not restricted to episodic and semantic memory functioning. Thus, results from the present study rather support the notion that the cognitive stimulation caused by social activity is related to hippocampal functioning.

Most prior studies investigating relations between social network size and cognitive performance have either used global measures of cognitive functioning (Bassuk et al. 1999; Glei et al. 2005; Holtzman et al. 2004) or composites including a range of different abilities (e.g., Barnes et al. 2004, Seeman et al. 2001a, b; Zunzunegui et al. 2003) and therefore have not generated knowledge concerning the potential differences along the fluid-crystallized continuum. Moreover, the only study that included a measure of social network size and a broader set of abilities (episodic memory, semantic memory, working memory, perception speed, and visuospatial ability) in an older population (Krueger et al. 2009) failed to establish associations with a global measure of cognitive function (i.e., sum of all domains) and therefore performed no further analysis of the specific cognitive domains. Hence, additional studies are needed to provide additional evidence of differential relations between social networks and specific cognitive ability factors.

One limitation of the present study is that we lacked sufficient longitudinal data to more extensively analyze the covariance of time-related changes in both cognition and network size, as only a few of the participants had longitudinal follow-up data on network size. However, we were able to perform cross-lagged panel correlation analyses. Even if causal directionality cannot be determined from such analyses involving two measurement waves (Kenny 1975), the results from our analyses of a subgroup of participants with longitudinal data for both constructs—indicating that T1 network size was associated with T3 episodic and semantic memory performance, whereas episodic and semantic ability at baseline were unrelated to future network size—are noteworthy. First, unequal cross-lagged correlations argue against the notion that a third confounding variable underlies the association (Kenny 1975). Second, the pattern seems to be in accordance with studies using more sophisticated methods and larger samples. For example, Lövdén et al. (2005) found that social participation (e.g., leisure and social activities) influenced subsequent change in perceptual speed, using a dual change score model, but no significant association was found in the opposite direction. In a similar vein, Ertel et al. (2008) found that higher baseline engagement in social relationships predicted slower memory decline (immediate and delayed recall), but there was no evidence of a reverse direction. Thus, the available data, although limited, seem consistent with a directional influence from social network size to cognitive performance, in line with these two studies (but see Aartsen et al. 2002).

In conclusion, the present results suggest that the size of an individual’s network may influence aspects of cognitive functioning such as episodic and semantic memory. Improving our understanding of the mechanisms through which social networks influence cognition requires further investigation. Future research should examine the generality of these patterns across ability factors other than those targeted in the present study. Finally, intervention studies may constitute a valuable supplement to correlational studies regarding the issue of the directionality of causal influences, as this issue has not yet been fully settled.
